# TIME TO EMBRACE HETEROGENEITY IN INTERVENTIONAL STUDIES OF OLDER ADULTS

**DOI:** 10.1093/geroni/igac059.710

**Published:** 2022-12-20

**Authors:** Qian-Li Xue

**Affiliations:** Johns Hopkins University, Baltimore, Maryland, United States

## Abstract

Adopting evidence-based medicine in clinical care of older patients is challenging because the “best” evidence available may not be directly applicable due to exclusion or underrepresentation of older adults in clinical trials. Interventions shown to be beneficial in trial populations therefore often exhibit heterogeneous treatment effects (HTEs) in older adults, particularly among the most vulnerable. This talk will review the concept, causes, and estimation of HTEs. We distinguish clinical heterogeneity from methodologic heterogeneity, defined respectively as the variation in biological mechanisms leading to similar aging phenotypes and variation in study design, measurement, and analysis. We use examples drawn from geriatric medicine to introduce novel study designs and data analytics used to study HTEs. This talk highlights the importance of moving beyond post-hoc subgroup analysis to an approach that integrates theories, observational and experimental data, and data science in the study of HTEs.

